# Teachers’ gestures and students’ learning: sometimes “hands off” is better

**DOI:** 10.1186/s41235-017-0077-0

**Published:** 2017-10-25

**Authors:** Amelia Yeo, Iasmine Ledesma, Mitchell J. Nathan, Martha W. Alibali, R. Breckinridge Church

**Affiliations:** 1 University of Wisconsin, Madison, Wisconsin USA; 2 Northeastern Illinois University, Chicago, Illinois USA

## Abstract

During mathematics instruction, teachers often make links between different representations of mathematical information, and they sometimes use gestures to refer to the representations that they link. In this research, we investigated the role of such gestures in students’ learning from lessons about links between linear equations and corresponding graphs. Eighty-two middle-school students completed a pretest, viewed a video lesson, and then completed a posttest comparable to the pretest. In all of the video lessons, the teacher explained the links between equations and graphs in speech. The lessons varied in whether the teacher referred to the equations in gesture and in whether she referred to the graphs in gesture, yielding four conditions: neither equations nor graphs, equations only, graphs only, and both equations and graphs. In all conditions, the gestures were redundant with speech, in the sense that the referents of the gestures were also mentioned in speech (e.g., pointing to “2” while saying “2”). Students showed substantial learning in all conditions. However, students learned less when the teacher referred to the equations in gesture than when she did not. This was not the case for gesture to graphs. These findings are discussed in terms of the processing implications of redundancy between gesture and speech, and the possibility of “trade-offs” in attention to the visual representations. The findings underscore the need for a more nuanced view of the role of teachers’ gestures in students’ comprehension and learning.

**Electronic supplementary material:**

The online version of this article (doi:10.1186/s41235-017-0077-0) contains supplementary material, which is available to authorized users.

## Significance statement

Teachers regularly produce gestures during instruction. These gestures have the potential to influence students’ learning. However, to date, few empirical studies have examined the effectiveness of different types of gestures in students’ mathematical learning, relative to appropriate controls. Mathematical instruction often involves making connections between two or more representations (e.g., an equation and a graph). Teachers’ gestures may influence students’ grasp of such connections. For example, if a teacher points sequentially to the intercept term in the equation and then to the intercept on the graph, this could influence students’ grasp of the connection between the graph and the equation. In this paper, we report basic research designed to investigate whether teacher gesture influences student learning from lessons about links between graphs and equations. Students showed substantial learning in all conditions. However, students learned less when the teacher referred to the equations in gesture than when she did not. This was not the case for gesture to graphs. Past research has shown that teachers can adjust their gestures when they wish to do so; therefore, these findings may need to be considered when attempting to improve instructional communication in mathematics.

## Background

### Teachers’ gestures and students’ learning: sometimes “hands off” is better

During mathematics instruction, teachers often make connections between different representations of mathematical information, and they sometimes use gestures to refer to the representations that they link. In this research, we investigated the role of such gestures in students’ mathematics learning.

Deep understanding of mathematics requires understanding of connections among ideas, including connections between concepts and procedures, connections among steps within procedures, and connections among different mathematical representations. Indeed “connections” is one of the standards for mathematics instruction described by the National Council of Teachers of Mathematics in their *Principles and Standards for School Mathematics* (NCTM, 2000), and the Common Core State Standards for mathematics also reflect the importance of connections among ideas (NGACBP & CCSSO, 2010; Koestler, Felton, Bieda, & Otten, 2013). Given the value of understanding connections in mathematics, it is not surprising that mathematics instruction often focuses on connecting ideas. Some researchers have called for an increased focus on connection-making in instruction as a means to promote mathematics achievement (Richland, Stigler, & Holyoak, 2012).

Teachers’ communication about connections among mathematical ideas may be crucial to students’ learning about them. Several previous studies have sought to document how teachers communicate about connections among ideas in instruction in classroom settings (Alibali et al., 2014; Richland, Zur, & Holyoak, 2007). These past studies have established that teachers often refer to the ideas being connected using both speech and gestures. For example, Richland et al. (2007, p. 1129) identified comparative gestures (e.g., “pointing back and forth between a scale and an equation”) as one technique that teachers use to support students’ understanding of instructional analogies, a common form of connection-making. In this research, we investigate whether student learning is best supported when teachers refer to the ideas being connected using gestures as well as speech.

Alibali et al. (2014) presented a taxonomy of ways in which teachers use gestures in communicating about connections during mathematics instruction. They identified episodes within lessons that involved connecting ideas, termed *linking episodes*, and examined whether teachers tended to refer to both (or all) of the linked ideas multi-modally (typically in speech and gesture, sometimes speech and writing), or whether teachers sometimes referred to linked ideas in one modality on its own (typically speech alone, although, in rare cases, teachers referred to a linked idea using gesture alone). The teachers in their sample typically referred to both (or all) of the linked ideas multi-modally; for example, one teacher linked the standard notation of a multi-digit number (206,895) with the corresponding expanded notation (2 · 10^5^ + 6 · 10^3^ + 8 · 10^2^ + 9 · 10^1^ + 5 · 10^0^) by sequentially pointing to each digit in the standard notation and then to its corresponding power of 10 in the expanded notation. However, there was substantial variation across teachers in how regularly they referred to the linked ideas multi-modally, with one teacher referring to both (or all) of the linked ideas multi-modally in only 65% of linking episodes, and another referring to both (or all) of the linked ideas multi-modally in 100% of linking episodes.

Thus, it is well established that teachers do communicate about links between mathematical ideas, and that they often do so multi-modally. However, little is known about whether variations in teachers’ communication about links actually make a difference for students’ learning. As such, there is little empirical basis for specific recommendations about practice.

In particular, little is known about whether variations in teachers’ gestures during linking episodes make a difference for students’ learning. A growing body of research suggests that, in general, teachers’ gestures during instruction are beneficial for mathematical learning. These studies have focused on a range of different concepts, including symmetry (Valenzeno, Alibali, & Klatzky, 2003), Piagetian conservation (Church, Ayman-Nolley, & Mahootian, 2004), and mathematical equivalence (Cook, Duffy, & Fenn, 2013; Koumoutsakis, Church, Alibali, Singer & Ayman-Nolley, 2016). However, no research to date has directly examined whether teachers’ gestures that refer to linked representations make a difference for students’ learning. Is it more beneficial for students when teachers refer to each of the linked representations multi-modally? Given that gestures contribute to language comprehension (Hostetter, 2011), it stands to reason that it would be beneficial for student learning when teachers gesture to each of the linked representations using both gesture and speech.

Why might we expect that referring to linked representations with gesture is beneficial for students’ learning? One clue comes from evidence about the types of gestures that teachers commonly use. Naturalistic studies have documented that teachers use a range of different types of gestures during instruction. Teachers frequently use pointing gestures to guide students’ attention to important features of the instructional context, such as key features of inscriptions (e.g., graphs, equations, or diagrams) that are written on the board (Alibali, Nathan, & Fujimori, 2011). Such gestures might facilitate students’ noticing and encoding the referents of those points. Teachers also regularly use pointing gestures when linking ideas. Sets of points can be used to highlight corresponding aspects of multiple representations, and in this way, to express information about relationships among ideas. For example, a teacher might point to the *y*-intercept of a linear equation, and then point to the corresponding *y*-intercept on the associated graph, or a teacher might point out the term that indicates slope in one equation, and then point out the corresponding term in a second equation. Pointing gestures can also clarify the referents of the accompanying speech, so they may contribute to students’ comprehension of links that are expressed verbally. For example, a teacher might say, “the *y*-axis represents the amount of money saved,” while pointing to the *y*-axis of the graph. In this case, her pointing gesture can clarify the referent of the term “*y*-axis” for students who are unsure.

In this experiment, we investigated how variations in teachers’ multi-modal reference to linked mathematical representations influenced students’ learning. To date, most research demonstrating the benefits of teachers’ gesture in instruction has focused on elementary school children’s learning of concrete mathematical concepts. Few studies have examined the role of gesture in middle-school children’s learning of more abstract mathematical concepts, for which linking representations may be particularly important. Therefore, we chose to examine the influence of teachers’ gestures on student learning in a middle-school mathematics lesson that focused on linear equations, and, specifically, on how slope and intercept are represented in symbolic equations and in graphs. The teacher’s speech was identical across lessons, but the teacher’s gestures varied so that we could test hypotheses about the effects of teachers’ gestures on students’ learning.

All of the lessons made connections between equations and graphs in speech; the lessons varied only in which representations the teacher referred to using gestures. We varied whether the teacher referred to the equations in gestures (or not), and whether the teacher referred to the graphs in gesture (or not), yielding four conditions: teacher gesture to both equations and graphs, teacher gesture to equations only, teacher gesture to graphs only, and teacher gesture to neither equations nor graphs. To assess learning, we developed assessment items that required students to understand connections among representations – that is, to generate a graph given an equation and/or a story, or to generate an equation given a graph and/or a story. We evaluated students’ success at representing both slope and intercept in the equations and graphs that they constructed. We hypothesized that there would be beneficial effects of referring to the equations in gestures, as well as beneficial effects of referring to the graphs in gestures. Moreover, we expected that these effects would be additive, such that gesture to both representations would be better than gesture to one representation only. We also considered the possibility that these effects might be interactive, with the combination leading to enhanced performance above and beyond the added effects of gesture to each representation on its own. Thus, we predicted that students would show the greatest learning in the condition in which the teacher referred to both the equations and the graphs with gestures and speech.

We expected that teachers’ pointing and tracing gestures would guide students’ attention to key features of the equations and graphs. As such, we expected that teachers’ gestures might also foster students’ learning to encode key features of those representations. Therefore, in addition to assessing students’ learning about links between representations, we also assessed students’ abilities to accurately encode equations and graphs. We hypothesized that there would be beneficial effects of referring to the equations in gestures on encoding of equations, and there would be beneficial effects of referring to graphs in gestures on encoding of graphs.

## Method

### Materials

#### Video lessons

The experiment utilized four video lessons of a female teacher providing a 20-min lesson on slope and intercept. All four lessons used the same verbal script and the same visual representations (i.e., graphs and equations). All four lessons also utilized the same audio track. We scripted the teacher’s use of gaze and gesture across the four lessons (see “Design,” below), based on previous research on teachers’ instruction about slope and intercept in more naturalistic settings (e.g., Alibali et al., 2013).

To construct the four lesson videos, the teacher first made an audio-recording of the lesson script before the lesson videos were filmed. During filming of each of the four videos, the teacher lip-synced to the audio track and produced the scripted gaze and gestures. Thus, the teacher’s speech was held constant across the four video lessons. The lessons varied only in her gesture and gaze, as described below.

#### Assessments of student knowledge about graphs and equations

We also developed a problem-solving assessment to tap students’ understanding of graphs and equations and the links between them. The items used on the problem-solving pretest and posttest were isomorphic in structure, but used different base equations and (for items that involved stories) different cover stories (see Additional file 1: Appendix A for examples). There were a total of 10 items on the pretest and 10 on the posttest.

Eight of the items at each test were *translation* items, which required students to translate between different representations of mathematical information (see Additional file 1: Appendix A). In four of these items, students were asked to generate a graph, in one case based on a story, in one case based on an equation, and in two cases, based on a story and an equation. In four of these items, students were asked to generate an equation, in one case based on a story, in one case based on a graph, and in two cases, based on both a story and a graph. In this paper, we refer to the type of output generated (i.e., graph or equation) as the *output representation*. We analyzed student performance on the translation items including output representation (i.e., graph or equation) as a within-participants predictor variable because we expected that students might differ in their abilities to generate graphs vs. equations, both before and after the lessons.

In addition to the eight translation items, there were two additional items at each test that were intended to tap students’ more general, conceptual understanding of slope and intercept and how they are represented in equations and graphs. In one of these items, students were asked simply to *identify the terms* that indicated slope and intercept in an equation. In the other item, students were asked to *write a sentence* explaining how the terms in an equation related to a corresponding graph (see Additional file 1: Appendix A). These items were analyzed separately from the translation items because output representation was not a relevant predictor of performance for these items.

#### Assessments of students’ encoding of graphs and equations

We also developed items to assess students’ encoding of graphs and equations (see Additional file 1: Appendix A). In these items, students were asked to draw a graph or to reproduce an equation that they viewed briefly. Students completed two equation-encoding items and two graph-encoding items at pretest and at posttest.

### Participants

Eighty-two 7th-grade students from a mid-sized city in the US Midwest participated in this study. The majority of the participants (*n* = 70) were drawn from public middle schools; the remainder were parochial- or private-school students recruited from a database of research participants maintained by the laboratory. The sample consisted of 34 female students and 48 male students. Thirty-five students were tested in the month before the beginning of their 7th-grade year (August), and 47 students were tested in the fall of their 7th-grade year (i.e., in September, October, or November). Based on student or parent self-report of demographic information, 78% of the students were White, 5% were Black, 4% were Asian, 4% were Hispanic, and 10% claimed more than one racial or ethnic category.

### Design

The experiment utilized a 2 (teacher gesture to graphs: yes or no) × 2 (teacher gesture to equations: yes or no) design which yielded four lesson conditions: no gesture, gesture to equations only, gesture to graphs only, or gesture to both equations and graphs. In all of the conditions, the teacher gazed to elements of the visual representations when she mentioned them; her gaze was scripted and did not vary across conditions. In the no-gesture condition, the teacher did not produce any hand gestures to the visual representations; however, she did gaze to elements of the graphs and equations when she spoke about them. In the gesture-to-graphs-only condition, the teacher pointed (and gazed) to elements of the graphs when she mentioned them during the lesson, but she did not point to the equations. Thus, she pointed and gazed to elements of the graphs, but only gazed to elements of the equations. In the gesture-to-equations-only condition, the teacher gestured to elements of the equations when she mentioned them during the lesson, but did not point to the graphs. Thus, she pointed and gazed to elements of the equations, but only gazed to elements of the graphs. In the gesture-to-both-graphs-and-equations condition, the teacher pointed (and gazed) to elements of both the graphs and the equations during the lesson when she mentioned them.

All of the scripted gestures were redundant with the co-expressive speech; for example, the teacher pointed to “y” in the equation while saying “y.” All of the information in the lesson was explicit in the teachers’ speech; the teacher’s gestures did not provide new information, but instead only highlighted the referents of the teacher’s speech. Thus, depending on condition, the teacher’s gesture served to reinforce the teacher’s reference either to both of the linked representations, to the graphs only, to the equations only, or to neither representation.

The speech-plus-gesture script for the lesson that included teacher gesture to both the equations and the graphs is presented in (Additional file 1). For the lesson that included only teacher gesture to the equations, the script was identical, with the exception that all gestures that referred to the graphs were omitted. Likewise, for the lesson that included only teacher gesture to the graphs, the script was identical, with the exception that all gestures that referred to the equations were omitted. Finally, for the lesson with gesture to neither the graphs nor the equations, all gestures were omitted.

### Procedure

Data were collected in groups of one to four students each. Each group was randomly assigned to view one of the four video lessons. At the outset of the session, parents provided consent for students to participate, and students provided assent to participate. Next, each student completed a pretest to assess baseline knowledge of slope and intercept. The pretest included two items to assess encoding of equations, two items to assess encoding of graphs, and 10 problem-solving items (8 translation items and 2 conceptual knowledge items) . Students were given 3 min to complete the encoding items and 12 min to complete the problem-solving items.

After the pretest, students watched a video lesson according to their assigned condition (no gesture, gesture to equations only, gesture to graphs only, or gesture to both equations and graphs). After watching the video, students completed a posttest, parallel to the pretest, to assess their encoding and problem solving following the lesson. At posttest, students were also given 3 min to complete the encoding items and 12 min to complete the problem-solving items.

### Coding

For both encoding items and problem-solving items, participants’ responses were coded for whether they correctly represented both slope and intercept. For items that required students to generate equations, slope responses were considered correct if the value provided as the slope in the equation was exactly correct, and intercept responses were considered correct if the value provided as the intercept in the equation was exactly correct. For items that required students to generate graphs, intercept responses were considered correct if the graph intersected the correct point on the *y*-axis. Slope responses were considered correct if a straight line calculated with the intercept and the last point of the graph had the correct gradient. For the item in which students were asked to identify the slope and intercept terms in an equation, responses were considered correct if they correctly identified the appropriate terms. For the item in which students were asked to write a sentence explaining how the terms in the equation related to the graph, slope responses were considered correct if they conveyed how the slope term in the equation related to the slope of the graph, and intercept responses were considered correct if they conveyed how the intercept term in the equation related to the intercept of the graph.

A second coder who was blinded to the participants’ condition assignments coded the responses of 13 randomly selected participants for accuracy. Agreement between coders was 86% for slope responses on encoding items, 97% for intercept responses on encoding items, 93% for slope responses on problem-solving items, and 98% for intercept responses on problem-solving items.

## Results

There were five categories of assessment items which were designed to assess students’ abilities to: (1) accurately encode equations (two items), (2) accurately encode graphs (two items), (3) translate among representations (eight items), (4) identify slope and intercept terms in an equation (one item), and (5) explain the relation between a graph and an equation in words (one item). Table 1 presents the average proportion correct for participants in each teacher gesture condition at pretest and posttest for each category of assessment items. Students’ performance at pretest did not vary significantly as a function of teacher gesture condition.

**Table 1:** Average proportion correct at pretest and posttest in each gesture condition and for each type of assessment item

		Gesture (G) condition							
		No G to equation				G to equation			
		No G to graph (*N* = 19)		G to graph (*N* = 20)		No G to graph (*N* = 20)		G to graph (*N* = 23)	
Item type	Number of items	Pre	Post	Pre	Post	Pre	Post	Pre	Post
Equation encoding	2	.88	.99	.88	.95	.86	.95	.83	.98
Graph encoding	2	.30	.51	.25	.61	.21	.48	.26	.55
Translation	8	.26	.64	.35	.76	.37	.59	.35	.64
Identify terms in equation	1	.11	.63	.15	.60	.15	.63	.22	.65
Describe relation in words	1	.13	.55	.20	.65	.15	.45	.09	.65

Note: For each item, two problem elements were coded (slope and intercept).

Our primary goal was to examine whether students’ posttest performance varied as a function of teacher gesture condition. To address this question, we analyzed the data using linear mixed effects models in the *lme4* R package (Bates, Maechler, Bolker, & Walker, 2014). For each outcome variable, we identified the model of best fit by starting with a model that included the factors we manipulated in the lessons (gesture to graphs, gesture to equations), and additional factors that characterized the items (element (intercept or slope) and, where applicable, output representation (graph or equation)), as well as potential interactions among these factors. We also included participants’ age (in months) and pretest performance on the corresponding items and elements. For each model, we started with a maximal random-effects structure (Barr, Levy, Scheepers, & Tily, 2013); we then examined the correlations among the random effects, and for correlations that were near 1, we simplified the random effects-structure to prevent the models being over-parameterized (Bates, Kliegl, Vasishth, & Baayen, 2015). For each outcome measure, we used model comparisons and evaluations of parameter estimates to identify the model of best fit. To test our hypotheses, we used model comparisons to evaluate the significance of hypothesized effects. In each case, these model comparisons involved comparing the log likelihoods of nested models with and without the term in question. For each outcome variable, we report the model of best fit, and we report model comparisons that test our hypotheses about teacher gesture to graphs and equations.

### Encoding performance

Participants performed well on the pretest equation-encoding items, but poorly on the pretest graph-encoding items (see Table 1). Thus, the equation items were quite easy for students, whereas the graph items were challenging. Because there was little room for improvement on the equation-encoding items, our analysis of students’ encoding performance focused on the graph-encoding items only. For each problem element (slope or intercept), we included pretest scores on the corresponding element as a potential predictor of posttest performance; these scores ranged from 0 to 2 as there were two pretest graph-encoding items.

**Fig. 1:**
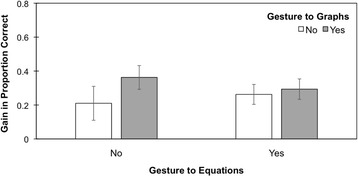
Average unadjusted gains in proportion correct on encoding graphs from pretest to posttest, as a function of condition. The error bars represent standard errors

The model of best fit for graph-encoding performance included only the main effect of problem element (slope or intercept) and random effects of participant and item (see Table 2 for details of the model specification). We had predicted that teacher gesture to graphs would promote students’ encoding of the graphs. To test this hypothesis, we compared a model that included teacher gesture to graphs with a model that included only problem element and the random effects. Although there was a trend in the expected direction, including teacher gesture to graphs did not significantly improve model fit, *χ* ^*2*^(1) = 2.81, *p* = .09, *OR* = 1.65, 95% confidence interval (CI) [0.93, 2.91]. Thus, contrary to our hypothesis, there was no evidence that teacher gesture to graphs promoted students’ accurate graph encoding (see Fig. 1 for raw, unadjusted gains in each condition). In similar model comparisons, we also found no evidence that teacher gesture to equations influenced student encoding of graphs, *χ* ^*2*^(1) = 0.79, *p* = .37, and no evidence for an interactive effect of gesture to graphs and gesture to equations, *χ* ^*2*^(1) = .042, *p* = .84.

**Table 2:** Model parameters for the best-fitting model for graph-encoding items

Fixed effects	Estimate	SE	*z* value	*p*
Intercept	0.95	0.76	1.24	0.21
Element	1.44	0.27	5.40	< .001
Random effects	Variance	SD		
Item	0.42	0.65		
Participant	1.08	1.04		

Posttest ~ Element + (1|item) + (1|participant). *SE* standard error, *SD* standard deviation

Students’ graph-encoding performance did vary across problem elements (slope vs. intercept), *z* = 5.40, *p* < .001, and including problem element (slope vs. intercept) in the model improved model fit, *χ* ^*2*^(1) = 30.20, *p* < .001. The odds of participants’ encoding intercept accurately were 4.23 times higher, 95% CI [2.51, 7.15] than the odds of their encoding slope accurately. These findings are sensible in light of the fact that correctly encoding intercept requires encoding only a single point, whereas correctly encoding slope requires encoding multiple points or a single point plus a slope value.

### Problem-solving performance

Recall that we predicted that there would be significant effects of teacher gesture to equations and teacher gesture to graphs on students’ problem solving. We further hypothesized that the combination of teacher gesture to equations and teacher gesture to graphs might have an interactive effect, yielding an additional boost in performance above and beyond the independent effects of gesture to equations and gesture to graphs on their own.

#### Translations among representations

We first examined student performance on the eight items that involved translations among representations. The model of best fit included significant main effects of teacher gesture to equations, problem element (slope or intercept), pretest performance (on the corresponding item and element), and age in months, as well as random effects of participant, item, and the slopes of output representation and element within participants (see Table 3 for details of the model specification). Including output representation as a main effect did not improve the fit of the model; students performed at similar levels whether they generated equations or graphs as the output of the problem-solving task, *χ* ^*2*^(1) = 0.39, *p* = .53. However, because the slope of output representation within participant as a random effect did improve model fit, we retained the main effect of output representation in the final model as well.

**Table 3:** Model parameters for the best-fitting model for translation items

Fixed effects	Estimate	SE	*z* value	*p*
Intercept	3.21	0.66	4.87	< .001
Gesture to equations	1.48	0.66	2.26	0.02
Age	0.18	0.08	2.29	0.02
Output	0.31	0.47	0.67	0.51
Element	1.99	0.28	7.15	< .001
Pretest	1.30	0.29	4.50	< .001
Random effects	Variance	SD		
Participant	14.54	3.81		
Element	2.10	1.45		
Output	3.59	1.89		
Item	0.25	0.50		

Posttest ~ Gesture to Equations + Age + Output + Element + Pretest + (1|item) + ((1 + Element + Output)|participant). *SE* standard error, *SD* standard deviation

None of the two-way or three-way interactions of the fixed effects significantly improved the fit of the model. Importantly, including the interaction of gesture to graphs and gesture to equations did not significantly improve model fit, *χ* ^*2*^(1) = 0.23, *p* = .63; thus, there was no evidence for an interactive effect of teacher gesture to graphs and teacher gesture to equations on translations among representations.

**Fig. 2:**
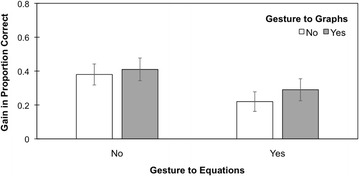
Average unadjusted gains in proportion correct on translation items from pretest to posttest, as a function of condition. The error bars represent standard errors

Contrary to our hypothesis that teacher gesture would enhance student performance, we found that students who received instruction that included teacher gesture to equations performed *more poorly* than students who received instruction that did not contain gesture to equations, *z* = −2.26, *p* = .02. Including gesture to equations in the model improved model fit significantly, *χ* ^*2*^(1) = 4.87, *p* = .03. The model estimated that the odds of correctly responding were 4.41 times higher, 95% CI [1.22, 15.96], among students who received instruction that did *not* include gesture to the equations than among students who received instruction that *did* include gestures to the equations. Thus, students who received lessons with gesture to the equations had lower posttest scores than those who received lessons without gesture to the equations. Figure 2 presents raw (unadjusted) pretest-to-posttest gains in each condition.

There was also no evidence that teacher gesture to graphs was beneficial for students’ performance at translating among representations. Although students who received instruction with gesture to graphs performed slightly better at posttest than those who received instruction without gesture to graphs, this effect was not significant, *χ* ^*2*^(1) = 2.81, *p* = .094, *OR* = 3.09, 95% CI [0.88, 10.84].

Students’ performance differed across problem elements, *z* = 7.15, *p* < .001, and including element (slope vs. intercept) in the model improved model fit, *χ* ^*2*^(1) = 34.19, *p* < .001. The model estimated that the odds of producing a correct response for intercept were 7.34 times, 95% CI [4.25, 12.68], the odds of producing a correct response for slope.

Not surprisingly, students performed better on items for which they had succeeded on the corresponding item at pretest than on items for which they had not succeeded on the corresponding item at pretest, *z* = 4.50, *p* < .001; including pretest scores in the model significantly improved model fit, *χ* ^*2*^(1) = 19.43, *p* < .001. The odds of producing a correct response at posttest were 3.66 times greater, 95% CI [2.08, 6.45], for items for which students had succeeded on the corresponding pretest item than for those on which they had not succeeded on the corresponding pretest item.

Students’ performance also varied as a function of age in months, *z* = −2.29, *p* = .02, and including age in the model significantly improved model fit, *χ* ^*2*^(1) = 4.75, *p* = .03. Surprisingly, however, the pattern was opposite to expectation; older students performed slightly less well on the posttest items than younger students. With each 1-month increase in age, the odds of producing a correct response at posttest declined by a factor of 0.83, 95% CI [0.71, 0.97].

#### Additional items

The two remaining items on the problem-solving test were intended to tap students’ more general, conceptual understanding of slope and intercept and how they are represented in equations and graphs. One item asked students to identify slope and intercept terms in an equation, and one asked students to describe relations between an equation and a corresponding graph in words. Data for each of these items individually are presented in Table 1; overall levels of performance were quite similar for the two items. For simplicity, we analyzed these two items together. The model of best fit included only pretest performance and the random effect of participant (see Table 4 for details of the model specification). Neither of the teacher gesture conditions nor their interaction significantly improved model fit: gesture to graphs, *χ* ^*2*^(1) = 0.72, *p* = .40; gesture to equations, *χ* ^*2*^(1) = 0.07, *p* = .79; the interaction of gesture to equations and gesture to graphs, *χ* ^*2*^(1) = 0.37, *p* = .55.

**Table 4:** Model parameters for additional items

Fixed effects	Estimate	SE	*z* value	*p*
Intercept	0.57	0.30	1.87	0.06
Pretest	2.27	0.68	3.33	< .001
Random effects	Variance	SD		
Participant	5.11	2.26		

Posttest ~ Pretest + (1|participant). *SE* standard error, *SD* standard deviation

## Discussion

### Empirical summary

In this research, we investigated whether teacher gestures to linked representations—including symbolic representations (equations) and visuospatial representations (graphs)—would promote student learning from a lesson about the links between those representations. Instead, we found that teachers’ gestures to the equations were detrimental to students’ learning. We did not find compelling evidence that teachers’ gestures to graphs were beneficial for students’ learning.

We also predicted that teachers’ gestures would lead students to better encode the representations that were the referents of those gestures. Students’ encoding of the equations was near ceiling, so we could test this hypothesis only for encoding of graphs. There was a trend that students who received teacher gesture to graphs encoded graphs more accurately at posttest than students who did not receive teacher gesture to graphs; however, this finding was not significant with alpha set at .05.

### Why were the teacher’s gestures to equations detrimental for student learning?

Why did students learn less when the teacher gestured to the equations? This finding flies in the face of abundant past research demonstrating that gesture contributes to comprehension of the accompanying speech (see Hostetter, 2011), and that teachers’ gestures are beneficial for students’ learning (e.g., Church et al., 2004; Cook et al., 2013).

In a meta-analytic review of whether gestures communicate, Hostetter (2011) identified several moderators of gesture’s effects on comprehension. These moderators might hold some clues about why gestures to equations were not beneficial in this study. One of these moderators was participants’ age; studies with participants younger than 12 years revealed greater communicative effects of gestures than studies with participants older than 12 years. Participants in the present study were between 11 and 13 years old; most were above the cut-off used to distinguish children and adults in Hostetter’s meta-analysis. Even so, this seems unlikely to be a major contributor to the present negative findings, as Hostetter reported a mean effect size for listeners aged 12 years and older that was substantial and positive, albeit smaller than the effect size for listeners younger than 12 years.

Hostetter (2011) also found that communicative effects of gesture were smaller when there was more overlap in the information expressed in gesture and speech, and larger when the information expressed in gesture and speech was non-redundant. Teachers sometimes express non-redundant information in gestures and speech (e.g., Goldin-Meadow, Kim & Singer, 1999), and in an experimental study, Singer and Goldin-Meadow (2005) found beneficial effects of teacher gestures on children’s learning about mathematical equivalence, but only for lessons in which the teacher’s gestures and speech were non-redundant, and not for lessons in which the teacher’s gestures and speech were redundant. These findings align with Hostetter’s (2011) conclusion that when speakers express redundant information in gesture and speech, gesture matters less for listeners’ comprehension.

Building on the findings of Singer and Goldin-Meadow (2005), Congdon et al. (2017) recently presented evidence that the *simultaneous* presentation of related but non-redundant information across modalities is particularly valuable for learning and transfer. They argued that simultaneous presentation of distinct information in speech and gesture encourages learners to actively integrate information across modalities; such integration is not necessary when information in the two modalities is redundant. From this perspective, gestures may need to add information in order to be beneficial.

In the present study, were the teacher’s gestures to the equations highly redundant with her speech? Consider the following utterance, which the teacher produced in the context of the equations *y* = 4*x*, *y* = 4 (2), and *y* = 8: “Let’s figure out what happens if James saves his money for 2 weeks. If we plug in 2 for *x*, we find that James will have saved 8 dollars because 4 times 2 is equal to 8.” In the conditions that included teacher gesture to the equations, the teacher pointed to the “2” when she said “2” in the phrase “if we plug in 2” and pointed to the “8” as she said “8 dollars.” In each case, her gesture was entirely redundant with her speech. Moreover, her speech alone would refer clearly, even in the absence of pointing gestures, because these digits are highly familiar to middle-school students, and because there was only one “2” and one “8” present in the visual representation. Thus, the teacher’s gestures did not include information that went beyond her speech.

The arguments discussed above offer a possible explanation for why the teacher’s gestures to the equations were not *beneficial* for student learning, but they cannot explain why the teacher’s gestures to the equations were actually *detrimental* for learning. What might account for the negative effect of the teacher's gesture to the equations that we observed? One possibility is that the redundancy of information in the teacher’s gestures and speech actually interfered with learning, in accord with Mayer’s “redundancy principle” for multi-media learning (see, e.g., Mayer, Heiser & Lonn, 2001). According to this principle, redundant information can evoke extraneous processing and thereby tax learners’ working memory capacity. Indeed, we insured that *all* of the teacher’s gestures in this study were redundant with the accompanying speech because we wanted to be able to examine whether that speech (in the no-gesture condition) would effectively communicate about links on its own. However, this feature of our design, while allowing us strong experimental control, also created a situation in which gesture was *always* redundant. From the perspective of Mayer’s cognitive theory of multi-media learning (e.g., Mayer, 2014), the redundant gestures may have evoked extraneous processing without contributing relevant, new information.

But, was the information in the teacher’s gestures to the equations actually *more* redundant than the information in the teacher’s gestures to the graphs? Perhaps so. As noted above, the teacher's speech referred quite unambiguously to the digits in the equations (e.g., pointing to the “2” in the equation while saying “2”), and in this sense, her gestures were redundant with her speech. The information expressed in gestures to the graph was also redundant with speech (e.g., the teacher said, “we go over to the 2 on the *x*-axis” while tracing along the *x*-axis from the origin to (0, 2)). However, there are inherent differences in how verbal and visuospatial representations present information; Schnotz (2002) characterized this difference in terms of the distinction between descriptive representations (such as text or equations), which represent content via convention, and depictive representations (visual displays, such as diagrams or graphs), which represent content via common structural features that are either concrete or abstract. One potential consequence of these differences is that gestures to visuospatial representations may be inherently less redundant with speech about those representations than are gestures to symbolic representations that occur with speech about those representations. If this is the case, then gestures to equations, by virtue of being more redundant with speech, should be more likely than gestures to graphs to evoke extraneous processing that can be detrimental to performance.

Another possibility has to do with the potential strangeness of the teacher’s communicative behavior in our lessons. Students may have found it strange for a teacher to gesture to equations (a symbolic representation) but not graphs (a visual representation), so the strangeness of the teacher’s communication in the gesture-to-equations-only condition may be partly responsible for the negative effect. However, performance in the gesture-to-both-equations-and-graphs condition was also poorer than performance in the gesture-to-graphs-only condition, arguing against this possibility.

A more likely possibility, in our view, is that teachers’ gestures to the equations may have encouraged students to attend to the equations *at the expense of* attending to the graphs. Since students were successful at encoding equations at the outset of the study, attending to the equations was not beneficial, and it may have reduced their attention to the more challenging graphical representations. The poorer encoding of graphs in the gesture-to-equations-only condition, relative to the gesture-to-graphs-only condition (see Fig. 1) is consistent with the possibility of such a “trade-off.” Future work that uses eye-tracking to monitor student attention might be valuable in testing this possibility.

In sum, there is a range of possible reasons that might explain why the teacher’s gestures to the equation were detrimental for students’ learning in this study. A trade-off in attention may be part of the reason; greater attention to the equation may lead to inadequate attention to the graphs. Alternatively, the greater redundancy between the information expressed in speech and in gestures to the equations may have evoked detrimental, extraneous processing, as suggested by Mayer’s redundancy principle.

The present negative findings are striking because very few published studies have reported null or negative effects of gestures on learning. One published study reports a null effect—specifically, on English-speaking adults’ learning of a vowel-length contrast in Japanese (Hirata & Kelly, 2010). Another recent study suggests potential negative effects of some types of gestures on listeners’ comprehension of speech in a spatial task (Suppes, Tzeng & Galguera, 2015), although it is not clear whether the negative effect in this study was due to the gesture or to the associated speech. Finally, one study of younger children learning about mathematical equations has reported a negative effect of learners’ own gestures on learning (Byrd, McNeil, D’Mello & Cook, 2014). It is also possible that other, past studies have yielded detrimental effects of teachers’ gestures, but these studies have gone unpublished (the “file drawer problem”). Indeed, Hostetter’s (2011) meta-analysis revealed that unpublished studies of communicative effects of gestures had a smaller mean effect size than did published studies, suggesting that some studies with null or negative effects may indeed be languishing in some researchers’ file drawers. Of course, regardless of whether the “file drawer problem” is actually a problem in this area, the current findings about the detrimental effects of teachers’ gestures to the equations are noteworthy, particularly in light of the careful controls implemented in this study.

### Limitations and future directions

The present findings underscore that many factors influence whether gestures are beneficial for communication, and, specifically, whether teachers’ gestures are beneficial for students’ learning. In this study, the effects of teachers’ gestures depended on the referents of those gestures. There was robust evidence that gestures to equations—a largely symbolic form of representation—were actually detrimental for student thinking. We also did not find evidence that gestures to graphs—a visuospatial form of representation—were helpful, although more definitive evidence on this point is needed. Future studies with greater power might provide more conclusive tests of the value of teacher gesture to graphs for students’ graph encoding and problem solving.

It is also worth noting that the present study included only two items to assess encoding of graphs, in an effort to keep the experimental session short, while also allowing time to assess other skills such as translating between representations. Future studies could focus more narrowly on graph encoding, using a wider range of items, as well as a larger sample of participants.

One open question is whether the present findings are limited to the specific context examined in this study—equations and graphs of linear functions—or whether they generalize to other situations in which teachers seek to link symbolic and visuospatial representations. To gain some leverage on the question of why gesture to the symbolic representation was detrimental to student learning, future research should compare the effects of teachers’ gestures to symbolic and visual representations, and to less familiar and more familiar representations. In addition, research should systematically investigate the effects of gestures that are more vs. less redundant with speech.

## Conclusion

Our findings highlight that it is not simply the case that more instructional gestures are better—some gestures may be unhelpful, and some may even be detrimental. In this study, less gesture—specifically, less gesture to the equations—was actually better for student learning. Thus, in making recommendations for teachers about how best to use gesture in their instruction, one should not simply encourage teachers to “gesture more.” Instead, our findings suggest that gestures may be especially valuable in communicating about visuospatial representations, particularly ones (like the graphs in this study) that are novel or unfamiliar. We suggest that teachers should use gestures in ways that highlight important features of visuospatial representations, and in ways that enrich and add to the information they express in speech.

At the broadest level, our findings underscore the need for a more nuanced view of the role of gesture in comprehension and learning. Teachers’ gestures are often beneficial, but this is not true in every case. A deeper understanding of the cognitive functions that gestures serve—both for speakers and for listeners—will help us to build richer theories that can explain, not only when and why gesture is beneficial for learning, but also when and why it is not.

## Additional file

Additional file 1: Appendix A. (DOCX 386 kb)
