# Supplementary material for: Teachers’ gestures and students’ learning: sometimes “hands off” is better
Source: Cogn Res Princ Implic. 2017 Oct 25;2:41. doi: 10.1186/s41235-017-0077-0 (PMC5655596; doi:10.1186/s41235-017-0077-0)
Supplement: Supplementary file 1 — Appendix A. (DOCX 386 kb) [file 41235_2017_77_MOESM1_ESM.docx]

Appendix A: test items and lesson script

1. Graph the following equation.

y = 2x + 3


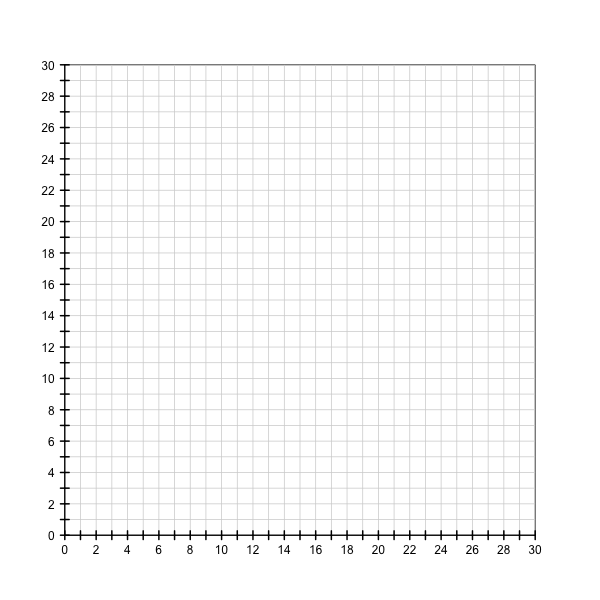


2. The graph of the equation, y = 3x + 6, is displayed below. Write a sentence explaining how the numbers in the equation relate to the graph.


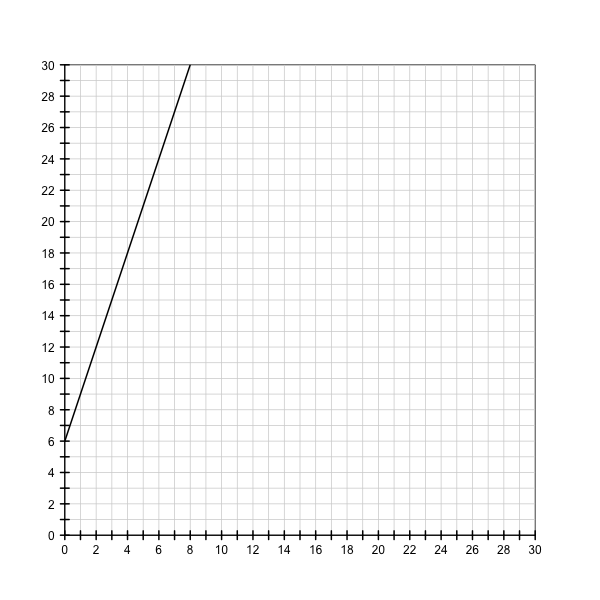


3. Matthew and Eric are making a picture book together. Matthew drew the pictures for the first 5 pages. Eric is going to draw the rest. He draws the pictures for 2 pages each day.

This story can be represented with the following equation:

y = 2x + 5

where y = the total number of pages

x = the number of days that Eric has drawn pictures

Draw a graph to represent this situation.


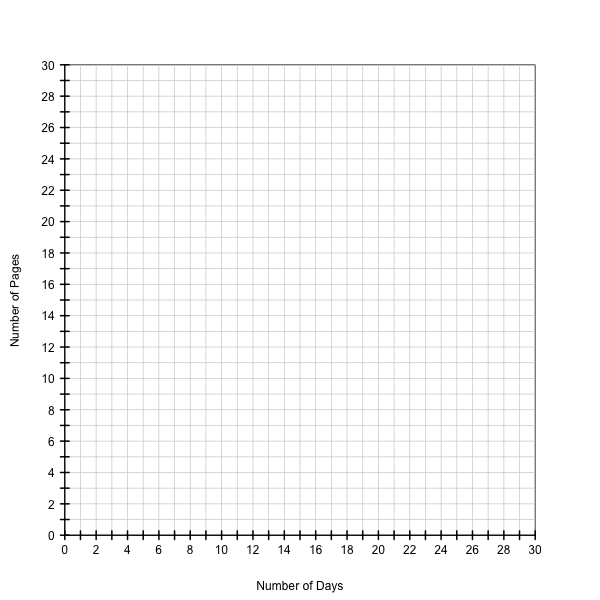


4. Andrew bought a bean plant at the garden store and planted it in his garden. It was 4 centimeters tall on the day he bought and planted it.

The graph below shows the growth of the plant, starting from when Andrew planted it.

Write an equation that represents the growth of Andrew’s plant.


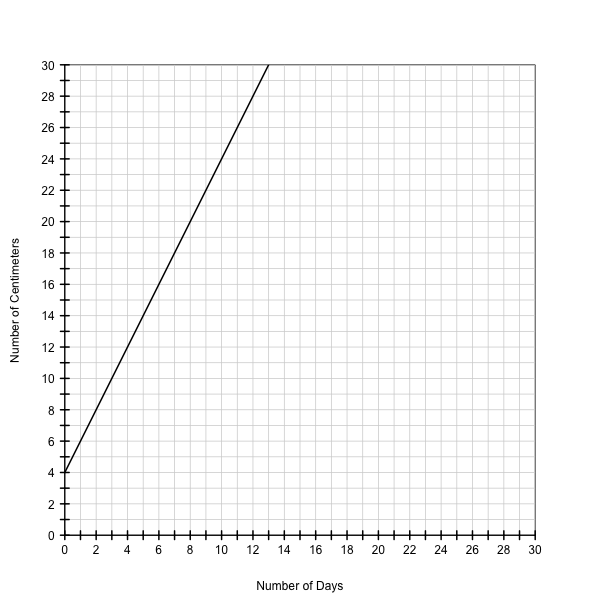


5. Chris has a new puppy, Blade. At birth, Blade weighed 8 ounces. Each day he gains 1 ounce.

Write an equation that represents this situation. Let y = Blade’s weight in ounces and let

x = the number of days since Blade was born.

6. Nicole is saving to buy an iPod. She starts out with $9 in her savings account and she saves $3 from her allowance each week.

Draw a graph that represents this situation.


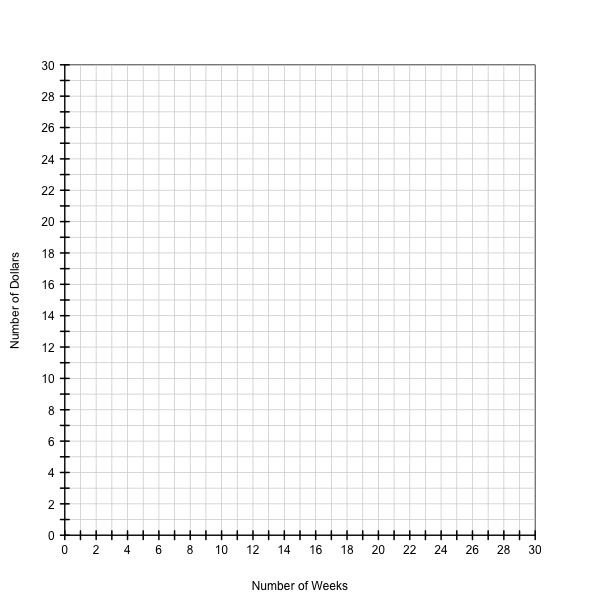


7. Write an equation for the line in the graph below.


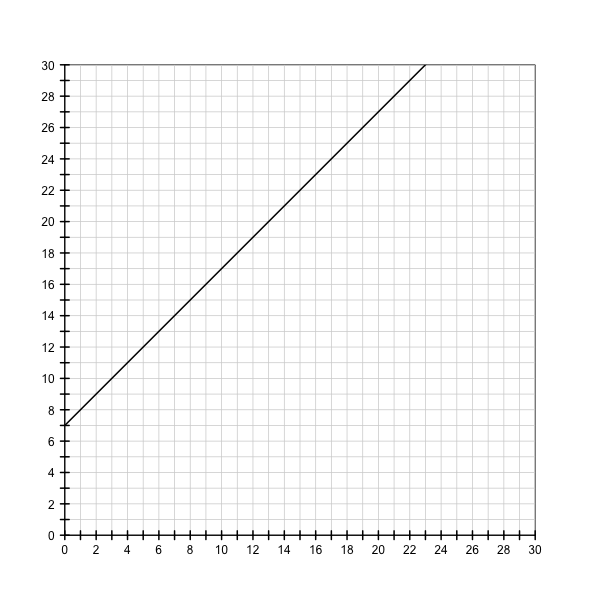


8. Katy is in a summer reading program at the public library. Students in the program get 3 points for each book they read. At the end of the summer, they receive prizes based on the number of points they have earned. At the beginning of the program, Katy gets 10 points as a bonus for getting a library card.

This story can be represented with the following equation:

y = 3x + 10

where y = the total number of points

x = the number of books Katy has read

Draw a graph to represent this situation.


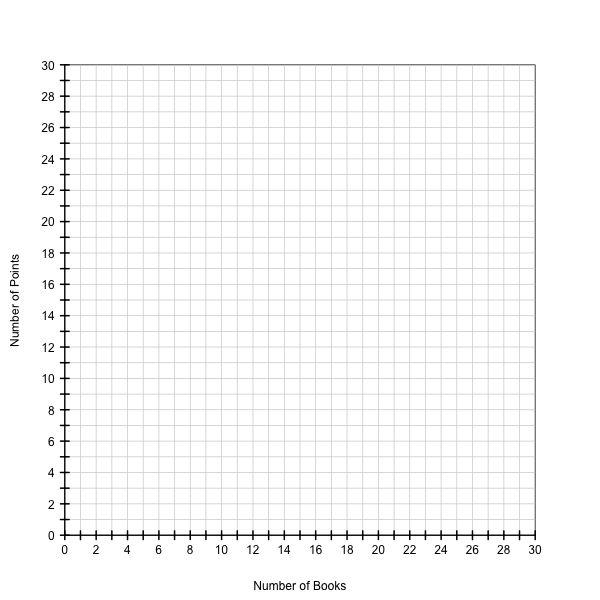


9. Eliza is collecting toy ponies. Her mom gave her 6 toy ponies to get her collection started. She uses her allowance to buy new ponies each week.

The graph below shows the size of Eliza’s toy pony collection, starting from when she received the 6 toy ponies from her mom.

Write an equation that represents the change in size of Eliza’s collection.


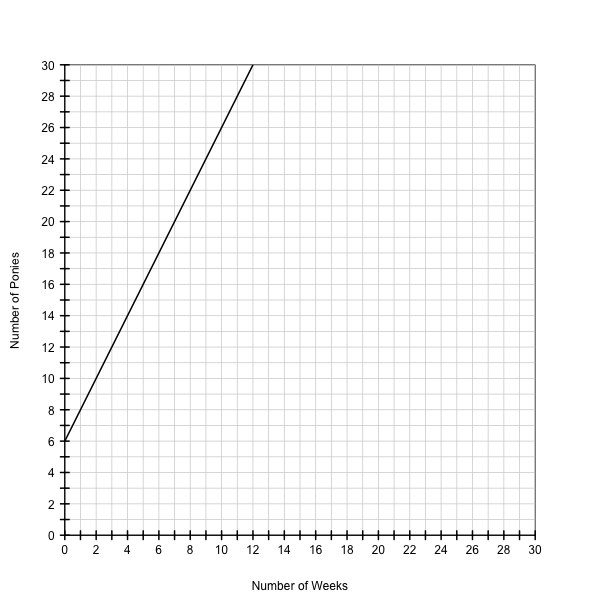


10. Consider the following equation:

y = 8x + 4

Complete the sentences below. You do not need to draw a graph.

The slope for the line of this equation is ______.

The y-intercept for the line of this equation is ______.

Lesson script

| Spoken | Slides |
| --- | --- |
| Today we are going to learn how to write equations and draw graphs to represent some stories! We will discover how we can present the same details in a variety of ways and how each of these ways can help us solve problems.  Imagine that your friend James is saving up to buy a bike and that he saves $4 each week from his allowance. How might we write out that situation as an equation? |  |
| How about: [dollars saved is equal to four dollars multiplied by the number of weeks. PALM]  So if we multiply [four dollars] by the number of [weeks] that James saves money, we can figure out the [total number of dollars] he saves in that time period! | Dollars saved = $4 x the # of weeks |
| To make the equation easier to read we can substitute letters, called variables, for some of our values. The equation now looks like this: [y equals four times x. PALM] | y = 4x |
| Remember that the [“y”] stands for the [total number of dollars] James saves and the [“x”] stands for the [number of weeks] he saves his money.  Variables can stand for any number. So, for example, if we plug in the number of weeks for [“x”] we can figure out [“y,”] or how many dollars James saved.  Let’s try plugging in some numbers! | y = 4x  Total dollars saved Weeks |
| So, if James saves his money for zero weeks, we can plug in [0] for x and then solve the equation. Four times zero equals zero [palm to third equation], so James will have saved a total of [zero dollars]. This makes sense because at zero weeks, James has not started to save money yet! | y = 4x  y = 4 (0)  y = 0 |
| Now imagine that James saves his money for one week. If we plug in [one] for x, we find that James will have saved [four dollars] because four times one is equal to four [palm to third equation]. | y = 4x  y = 4 (1)  y = 4 |
| Let’s figure out what happens if James saves his money for two weeks. If we plug in [two] for x, we find that James will have saved [eight dollars] because four times two is equal to eight [palm to third equation]. | y = 4x  y = 4 (2)  y = 8 |
| What if James saves his money for [three] weeks? Well four times three is equal to twelve [palm to third equation]. So James will have saved [twelve dollars]. | y = 4x  y = 4 (3)  y = 12 |
| Remember that we can plug in any number for x, so let’s try a bigger number! What happens if James saves his money for ten weeks? If we plug in [ten] for x, we find that James will have saved [forty dollars] because four times ten is equal to forty [palm to third equation]. | y = 4x  y = 4 (10)  y = 40 |
| Now we can use the values we just calculated to create a graph of the situation from our story! Making a graph can help us visualize the details. | y = 4x  0 = 4 (0)  4 = 4 (1)  8 = 4 (2)  12 = 4 (3)  40 = 4 (10) |
| Let’s look at the empty graph before we plot any points. The line going across the bottom is called the [“x-axis.” SWEEP] This line will help us in putting the “x” values from our equation - the number weeks that James saves his money - onto the graph. Remember that because the “x” stands for the number of weeks, the bottom line on the graph can be labeled as [“ Number of Weeks.”]  The vertical line on the left side of the graph is called the [“y-axis.” SWEEP] Remember that our “y” variable stands for the dollars that James saves, so the side of the graph can be labeled [“Dollars Saved.”] | Graph 1: Labeled axes with no plotted points |
| Now we can plot some different values for weeks and see how it affects the money James saves. If we look at our first equation, we remember that if James saves for [zero weeks], he will have saved [zero dollars]. So on our x –axis we start at the zero [place finger on (0,0, hold)] and on our y-axis we start at zero [place other finger on (0,0), hold], and then we can plot our first data-point at zero weeks and zero dollars!  We can use the same method to plot the rest of the values from our equations. | Graph 2: Labeled axes with point at 0,0  y = 4 (0) = 0 |
| If James saves for [one week], he will have saved [four dollars]. So we go over to the one on the x-axis [move finger from origin to (1,0), hold] and up four on the y-axis [move other finger from origin to (0,4), hold] and make a point [move fingers from their locations to the data point] at one week and four dollars. | Graph 3: Labeled axes with points at (0,0) and (1,4)  y = 4 (1) = 4 |
| If James saves for [two weeks], he will have saved [eight dollars]. So we go over to the two on the x-axis [move finger from origin to (2,0), hold] and up eight on the y-axis [move finger from origin to (0,8), hold] and make a point [move fingers from their locations to the data point] at two weeks and eight dollars. | Graph 4: Labeled axes with points at (0,0), (1,4) and (2,8)  y = 4 (2) = 8 |
| If James saves for [three weeks], he will have saved [twelve dollars]. So we go over to the three on the x-axis [move finger from origin to (3,0), hold] and up twelve on the y-axis [move finger from origin to (0,12), hold] and make a point [move fingers from their locations to the data point] at three weeks and twelve dollars. | Graph 5: Labeled axes with points at (0,0), (1,4), (2,8), and (3,12)  y = 4 (3) = 12 |
| If James saves for [ten weeks], he will have saved a total of [forty dollars]. So we go all the way over to the ten on the [x-axis SWEEP] and up to forty on the [y-axis SWEEP] and make a [point] at ten weeks and forty dollars. | Graph 6: Labeled axes with points at (0,0), (1,4), (2,8), (3,12), and (10,40)  y = 4 (10) = 40 |
| If we connect all the points we made, you will notice that they make a [line]! If we look at our graph, we see that for every one unit that we move over on the x-axis [move finger over 1 unit on x-axis, hold it there], we move up four units on the y-axis [move finger up 4 units]. We can say that this line has a slope of 4 because James’ money increases at a rate of 4 dollars per week. If we look back at our equation for this story, [y = 4x PALM], we see that the slope, [4], is the number we multiplied x by. The slope stays the same for the entire line, so if we move over one unit on the x-axis from 2 weeks to 3 weeks [move finger over 1 unit 2 to 3, hold it there], we then move up 4 units on the y-axis from 8 dollars to 12 dollars [move finger up 4 units from 8 to 12.]  We can also see that our line begins at [0] on the y-axis because at that point James has not saved any money yet. We call this point the y-intercept because it is the point at which our line hits the [y-axis SWEEP]. | Graph 7: Labeled axes with line connecting previously plotted points  y = 4x |
| Now let’s imagine that James doesn’t want to wait so long to save up enough money for his bike. So instead of saving four dollars from his allowance, he decides to save six dollars from his allowance each week. | y = 6x |
| So now our equation will look like this: [y equals six x] | y = 6x |
| Remember that the [“y”] stands for the [total dollars] James saves and the [“x”] stands for the number of [weeks] he saves his money. | y = 6x  Total dollars saved Weeks |
| If we think back to the first story where James saved only four dollars a week, we notice that the equations for the two situations look very similar, only the number being multiplied by the x has changed. In our new we multiply the x by [6] and in our old equation we multiply the x by [4]. This makes sense because this number represents the dollars that James saves each week, which is the only thing that has changed from our first story to our second.  So let’s see what happens when we plug some numbers into our new equation! | y=6x  y=4x |
| So, if James saves his money for zero weeks, we can plug in [zero] for x and then solve the equation. Six times zero equals zero [palm to third equation], so James will have saved a total of [zero dollars]. This makes sense because at zero weeks he hasn’t started saving money yet! | y = 6x  y = 6 (0)  y = 0 |
| Now imagine that James saves his money for one week. If we plug in [one] for x, we find that James will have saved [six dollars] because six times one is equal to six [palm to third equation]. | y = 6x  y = 6 (1)  y = 6 |
| Let’s figure out what happens if James saves his money for two weeks. If we plug in [two] for x, we find that James will have saved [twelve dollars] because six times two is equal to twelve [palm to third equation]. | y = 6x  y = 6 (2)  y = 12 |
| What if James saves his money for [three weeks]? Well six times three is equal to eighteen [palm to third equation], so James will have saved [eighteen dollars]. | y = 6x  y = 6 (3)  y = 18 |
| Remember that we can plug in any number for x, so let’s try a bigger number! What happens if James saves his money for ten weeks? If we plug in [ten] for x, we find that James will have saved [sixty dollars] because six times ten is equal to sixty [palm to third equation]. | y = 6x  y = 6 (10)  y = 60 |
| Now we can plot some points to make a graph for this new situation. | y = 6x  y = 6 (0) = 0  y = 6 (1) = 6  y = 6 (2) = 12  y = 6 (3) = 18  y = 6 (10) = 60 |
| If we look at our first equation, we remember that if James saves for [zero weeks], he will have saved [zero dollars]. So on our x –axis we start at the zero [place finger at (0,0), hold] and on our y-axis we start at zero [place other finger at (0,0), hold], and then we can make a point at zero weeks and zero dollars!  We can use the same method to plot the rest of the values. | Graph 8: Labeled axes with point at (0,0)  y = 6 (0) = 0 |
| If James saves for [one week], he will have saved [six dollars]. So we go over to the 1 on the x-axis [move finger from origin to (1,0), hold] and up 6 on the y-axis [move finger from origin to (0,6), hold] and make a point [move fingers from their locations to the data point] at one week and six dollars. | Graph 9: Labeled axes with points at (0,0) and (1,6)  y = 6 (1) = 6 |
| If James saves for [two weeks], he will have saved [twelve dollars]. So we go over to the two on the x-axis [move finger from origin to (2,0), hold] and up twelve on the y-axis [move finger from origin to (0,12), hold] and make a point [move fingers from their locations to the data point] at two weeks and twelve dollars. | Graph 10: Labeled axes with points at (0,0), (1,6), and (2,12)  y = 6 (2) = 12 |
| If James saves for [three weeks], he will have saved [eighteen dollars]. So we go over to the three on the x-axis [move finger from origin to (3,0), hold] and up eighteen on the y-axis [move finger from origin to (0,18), hold] and make a point [move fingers from their locations to the data point] at three weeks and eighteen dollars. | Graph 11: Labeled axes with points at (0,0), (1,6), (2,12), and (3,18)  y = 6 (3) = 18 |
| If James saves for [ten weeks], he will have saved a total of [sixty dollars]. So we go all the way over to the ten on the [x-axis SWEEP] and up to sixty on the [y-axis SWEEP] and make a [point] at ten weeks and sixty dollars. | Graph 12: Labeled axes with points at (0,0), (1,6), (2,12), (3,18), and (10,60)  y = 6 (10) = 60 |
| If we connect all the points, you will notice that they also make a [line]! If we look at our graph, we see that for every one unit that we move over on the x-axis [move finger over 1 unit on x-axis, hold it there], we move up six units on the y-axis [move finger up 6 units]. This line has a slope of 6 because James’ money increases at a rate of 6 dollars per week. If we look back at our equation for this story, [y = 6x PALM], we see that the slope, [6], is the number we multiplied x by. The slope stays the same for the entire line, so if we move over one unit on the x-axis from 2 weeks to 3 weeks [move finger over 1 unit 2 to 3, hold it there], we then move up 6 units on the y-axis from 12 dollars to 18 dollars [move finger up 4 units from 12 to 18.]  We can also see that our line has a y-intercept of [0] because at that point James has not saved any money yet. | Graph 13: Labeled axes with line connecting previously plotted points  y = 6x |
| Now let’s compare the lines on the graph from our two stories!  Remember that in one of our stories James was saving [four] dollars a week from his allowance and in the other story James was saving [six] dollars from his allowance each week. If we look at the graphs of the our stories, we notice that although they both make straight lines, they look a bit different. This is because the two lines increase at different rates, or have different slopes. This [equation PALM], when James was saving [four dollars] a week, has a slope of four because for every one unit we move over on the x-axis [move finger over 1 unit on x-axis, hold it there], we move up four units on the y-axis [move finger up 4 units]. If we look back at our equation for this story, [y = 4x PALM], we see that the slope, [4], is the number we multiplied x by. This equation, when James was saving [six] dollars a week, has a slope of six because for every one unit that we move over on the x-axis [move finger over 1 unit on x-axis, hold finger there], we move up six units on the y-axis [move finger up 6-units]. Again if we look at our equation for this story, [y = 6x PALM], we see that our slope of [six] is the number we multiplied by x. Notice that the line with the larger slope is steeper [point to end of steeper line]. This makes sense because James saved money faster when he saved six dollars a week than when he saved four dollars.  Although they have different slopes, both lines have the same y-intercept of [0]. This is because in both of the stories James had not saved any money yet at this point. | Graph 14: Labeled axes with lines of both equations (y=4x and y=6x). Equations will be labeled on graph. |
| Now let’s imagine that James’ grandmother gives him ten dollars to start off his savings. James still saves $6 from his allowance each week. What will our equation look like now?  Since James is still saving six dollars each week we can start off with the same equation: [y is equal to six times x PALM] | y = 6x |
| To show that his grandmother gave him ten dollars to start his savings, we simply add those on at the end of the equation: [y is equal to six times x plus ten. PALM]  Notice that the number we multiply by x stays the same because James is still saving [six] dollars per week. | y = 6x + 10 |
| Remember that the [“y”] stands for the [total dollars] James saves and the [“x”] stands for the number of [weeks] he saves his money.  Let’s try plugging in some numbers! | y = 6x + 10  Total dollars saved Weeks |
| So, if James saves his money for zero weeks, we can plug in [zero] for x and then solve the equation. Six times zero equals zero and then we add the ten dollars from his grandmother [palm to third equation]. and we find that James will have a total savings of [ten dollars]. This makes sense, he starts off with the gift from his grandmother but doesn’t save any money other than that! | y = 6x + 10  y = 6 (0) + 10  y = 10 |
| Now imagine that James saves his money for one week. If we plug in [one] for x, we find that James will have saved [sixteen] dollars because six times one is equal to six and six plus ten is equal to sixteen [palm to third equation]. | y = 6x + 10  y = 6 (1) + 10  y = 16 |
| Let’s figure out what happens if James saves his money for two weeks. If we plug in [two] for x, we find that James will have saved [twenty-two] dollars because six times two is equal to twelve and twelve plus ten is equal to twenty-two [palm to third equation]. | y = 6x + 10  y = 6 (2) + 10  y = 22 |
| What if James saves his money for [three weeks]? Well six times three is equal to eighteen [palm to third equation], and if we add the ten his grandmother gave him we find that James will have saved [twenty-eight] dollars. | y = 6x + 10  y = 6 (3) + 10  y = 28 |
| Remember that we can plug in any number for x, so let’s try a bigger number! What happens if James saves his money for ten weeks? If we plug in [ten] for x, we find that James will have saved [seventy] dollars because six times ten is equal to sixty and sixty plus ten is equal to seventy [palm to third equation]. | y = 6x + 10  y = 6 (10) + 10  y = 70 |
| Now we can plot some points to make a graph for the situation in our new story. | y = 6x + 10  y = 6 (0) + 10 = 10  y = 6 (1) + 10 = 16  y = 6 (2) + 10 = 22  y = 6 (3) + 10 = 28  y = 6 (10) + 10 = 70 |
| If we look at our first equation, we remember that if James receives [ten dollars] from his grandma and then saves for [zero weeks], he will have saved ten dollars. So on our x –axis we start at the zero [place finger at (0,0), hold] and on our y-axis we go up ten [move finger from origin to (0,10), hold], and then we can make a point [move fingers from their locations to the data point] at zero weeks and ten dollars!  We can use the same method to plot the rest of the values. | Graph 15: Labeled axes with point at (0,10)  y = 6 (0) + 10 = 10 |
| If James saves for [one week], he will have saved [sixteen dollars]. So we go over to the one on the x-axis [move finger from origin to (1,0), hold] and up sixteen on the y-axis [move finger from origin to (0,16), hold] and make a point [move fingers from their locations to the data point] at one week and sixteen dollars. | Graph 16: Labeled axes with points at (0,0) and (1,16)  y = 6 (1) + 10 = 16 |
| If James saves for [two weeks], he will have saved [twenty-two dollars]. So we go over to the two on the x-axis [move finger from origin to (2,0), hold] and up twenty-two on the y-axis [move finger from origin to (0,22), hold] and make a point [move fingers from their locations to the point] at two weeks and twenty-two dollars. | Graph 17: Labeled axes with points at (0,0), (1,16), and (2,22)  y = 6 (2) + 10 = 22 |
| If James saves for [three weeks], he will have saved [twenty-eight dollars]. So we go over to the three on the x-axis [move finger from origin to (3,0), hold] and up twenty-eight on the y-axis [move finger from origin to (0,28), hold] and make a point [move fingers from their locations to the data point] at three weeks and twenty-eight dollars. | Graph 18: Labeled axes with points at (0,0), (1,16), (2,22), and (3,28)  y = 6 (3) + 10 = 28 |
| Finally, if James saves for [ten weeks], he will have saved a total of [seventy dollars]. So we go all the way over to the ten on the [x-axis SWEEP] and up to seventy on the [y-axis SWEEP] and make a [point] at ten weeks and seventy dollars. | Graph 19: Labeled axes with points at (0,0), (1,16), (2,22), (3,28), and (10,70)  y = 6 (10) + 10 = 70 |
| If we connect all the points, you will notice that they also make a [line]! If we look at our graph, we see that for every one unit that we move over on the x-axis [move finger over 1 unit on x-axis, hold it there], we move up six units on the y-axis [move finger up 6 units]. This line has a slope of 6 because James’ money increases at a rate of 6 dollars per week. If we look back at our equation for this story, [y = 6x + 10 PALM], we see that the slope, [6], is the number we multiplied x by. The slope stays the same for the entire line, so if we move over one unit on the x-axis from 2 weeks to 3 weeks [move finger over 1 unit 2 to 3, hold it there], we then move up 6 units on the y-axis from 22 dollars to 28 dollars [move finger up 4 units from 22 to 28.]  We can also see that our line has a y-intercept of [10] because at that point James has the [ten dollars] he received from his grandmother, but has not saved any money other than that. | Graph 20: Labeled axes with line connecting previously plotted points  y = 6x + 10 |
| Remember that in one of our stories James was saving [six] dollars a week from his allowance and in the other story James still saved [six] dollars a week but also received a [ten] dollar gift from his grandmother. If we compare the graphs of our equations, we notice that they both have the same slope [palm to both lines]. For each one unit that we move over on the x-axis [move finger 1 unit over on the x-axis, hold finger there], we move up six units on the y-axis [move finger up 6 units] because James was always saving six dollars per week. If we look back at our equations we notice that in both we multiplied our x by [six]. However, the lines start in different places [sweep y-axis]! Why does this happen? This happens because the in our new equation, even when James doesn’t save any money on his own, he still starts with the [ten dollars] his grandmother gave him. These lines have different y-intercepts because they hit the y-axis at different points [sweep y-axis]. This [palm to y = 6x] equation has a [y-intercept of zero] and this equation [palm to y = 6x+10] has a [y-intercept of ten.] | Graph 21: Labeled axes with lines of both equations (y=6x and  y = 6x + 10).  Equations will be labeled on graph. |
| So today we have learned how we can represent the same stories with both mathematical equations and graphs.  We also learned that a line’s slope tells us about the steepness of the line [sweep to lines]. In our stories today, the slopes of the lines showed us how quickly the amount of money James saved increased.  Additionally we learned that a line’s y-intercept is the point at which the line hits the [y-axis SWEEP]. In our stories today, the y-intercepts of the lines showed us how much money James started out with each time. | Graph 22: Labeled axes with lines of all three equations. |
